# Dual-Stream Contrastive Latent Learning Generative Adversarial Network for Brain Image Synthesis and Tumor Classification

**DOI:** 10.3390/jimaging11040101

**Published:** 2025-03-28

**Authors:** Junaid Zafar, Vincent Koc, Haroon Zafar

**Affiliations:** 1 Faculty of Engineering, Government College University, Lahore 54000, Pakistan; chairperson.engineering@gcu.edu.pk; 2 Independent Researcher, Kuala Lumpur 55100, Malaysia; vincentkoc@ieee.org; 3 Lambe Institute for Translational Research, University of Galway, H91 YR71 Galway, Ireland

**Keywords:** GANs, tumor classification, brain imaging, MRI

## Abstract

Generative adversarial networks (GANs) prioritize pixel-level attributes over capturing the entire image distribution, which is critical in image synthesis. To address this challenge, we propose a dual-stream contrastive latent projection generative adversarial network (DSCLPGAN) for the robust augmentation of MRI images. The dual-stream generator in our architecture incorporates two specialized processing pathways: one is dedicated to local feature variation modeling, while the other captures global structural transformations, ensuring a more comprehensive synthesis of medical images. We used a transformer-based encoder–decoder framework for contextual coherence and the contrastive learning projection (CLP) module integrates contrastive loss into the latent space for generating diverse image samples. The generated images undergo adversarial refinement using an ensemble of specialized discriminators, where discriminator 1 (D1) ensures classification consistency with real MRI images, discriminator 2 (D2) produces a probability map of localized variations, and discriminator 3 (D3) preserves structural consistency. For validation, we utilized a publicly available MRI dataset which contains 3064 T1-weighted contrast-enhanced images with three types of brain tumors: meningioma (708 slices), glioma (1426 slices), and pituitary tumor (930 slices). The experimental results demonstrate state-of-the-art performance, achieving an SSIM of 0.99, classification accuracy of 99.4% for an augmentation diversity level of 5, and a PSNR of 34.6 dB. Our approach has the potential of generating high-fidelity augmentations for reliable AI-driven clinical decision support systems.

## 1. Introduction

Brain tumors (BTs) remain a significant public health concern, ranking as the 10th leading cause of death in the United States alone [1]. Research indicates that brain tumors are significantly impacting patients’ lives through physical, cognitive, and psychological impairments [2]. BTs can be benign or malignant, with benign tumors being slow-growing and localized, while malignant tumors are highly aggressive with metastasis [3]. Brain tumors (BTs) encompass a diverse range of types with varying degrees of aggressiveness, including glioblastoma, the most aggressive form; meningioma, which is often benign; and pituitary adenomas [4,5]. The World Health Organization (WHO) classifies brain tumors on a scale from Grades I to IV based on their degree of spread, biological behavior, and prognosis [6]. Targeted efforts are essential to enhance early detection methods and deepen the understanding of brain tumor progression, as early diagnosis expands treatment options and improves survival rates [7].

Magnetic resonance imaging (MRI) is a highly effective imaging modality for detecting and characterizing various aspects of brain tumors. It offers superior soft tissue contrast while minimizing patient exposure to ionizing radiation [8]. However, BT diagnosis through MRI scans is highly time-intensive and heavily dependent on the radiologist’s expertise. Accurately labeling the scans without misclassification presents challenges in both time and precision. To address this issue, various computer-aided solutions have been developed to support automated decision-making systems [9,10]. Artificial Intelligence (AI) has emerged as a promising tool for the early detection of BTs and is being leveraged to enhance diagnosis by analyzing MRI scans. Various deep learning architectures have been documented in the literature for the detection and classification of BTs using MRI scans [11,12,13,14,15]. Prakash et al. [16] presented an automated brain tumor classification method using an enhanced deep learning approach with DenseNet121. Transfer learning was applied, and hyper-parameter tuning optimized the CNN model. MRI images of three distinct brain tumor types were analyzed using the DenseNet169 model for feature extraction. The extracted features were then fed into three multi-class machine learning classifiers, Random Forest (RF), Support Vector Machine (SVM), and XGBoost, to enhance performance [17]. Elsewhere [18], the effectiveness of deep transfer learning has been assessed using ResNet152, VGG19, DenseNet169, and MobileNetv3 models. Similarly, Katkam et al. [19] presented the CapsNet model for multi-class classification of neuro-degenerative diseases using a modified DenseNet-169 framework coupled with the Enhanced DeepLab V3+ model. Further, a brief summary of seven deep learning models for BTs detection including VGG-16, VGG-19, ResNet50, Inception, ResNetV2, InceptionV3, Xception, and DenseNet201 and five traditional classifiers considering SVMs, RF, Decision Trees, AdaBoost, and Gradient Boosting are documented in the literature [20]. Although these advanced architectures have achieved promising results, they largely overlook the inherent limitations of imaging datasets, particularly significant class imbalances. The challenge of acquiring large, diverse datasets encompassing patients at various disease stages constrains the full potential of deep learning networks.

To overcome the challenges posed by limited medical imaging datasets, image augmentation techniques help in enriching data diversity, mitigating class imbalance, and improving model generalization. Adversarial learning frameworks have been employed to optimize the interaction between the generator and discriminator for creating high-quality synthetic medical images. In this regard, an automatic data GAN was used to learn the available annotated MRI samples of the BRATS15 Challenge dataset [21], and inception, resnetv2, inceptionv3, transfer learning, and BRAIN-TUMOR-net were published in [22] and used for MRI images of glioma tumors, meningiomas, and pituitary BTs. Han et al. [23] reported progressive GANs (PGGANs), in which multistage generative training was used to generate BT images that were challenging for conventional GANs, and further detailed reviews are available in [24,25,26,27,28] concerning the standard used augmentation methods and fusion deep learning models, including U-Net.

Transformers and auto-encoder architectures are imperative for MRI image augmentations due to their ability to overcome key limitations of deep learning CNN models, including long-range dependencies and global contextual awareness. A cross-transformer was published to include self-care model keys, queries, and values for the classification of BTs in MRI images and compared the results with InceptionResNetV2, InceptionV3, DenseNet121, Xception, ResNet50V2, VGG19, and EfficientNetB7 networks [29]. In [30], the possibilities of vision transformers (ViTs) being used as a viable alternative to CNNs for brain magnetic resonance imaging was pitched by incorporating self-attention mechanisms to establish relationships between image patches for a comprehensive understanding. Likewise, Zakariah et al. [31] highlighted the relevance of a dual vision transformer model (DSUNET) in providing reliable and efficient differentiation between brain tumors and other brain regions by leveraging the MRI BRATS 2020 dataset. In addition, a Swin transformer was introduced into the UNet++ network, in which the local features of BTs were extracted by a convolutional layer in UNet++ and global resolutions were captured via the shift window operation of the Swin transformer [32]. Researchers deployed a shrinking linear time vision transformer (SL(t)-ViT) network for enhanced classification across multiple datasets of BTs [33]. Hybrid architectures combined with cross-fusion allows parallel systems to be merged between branches, resulting in the reliable prediction of various tumors; similar studies are available in the literature [34,35]. In these studies, either a two-branch parallel model that integrated a transformer module (TM) with a self-attention mechanism was used to classify brain tumors in MR images or hybrid shifted window multi-head modules were deployed. To address the challenge of explainability in adversarial transformer models, graph attention network (GAT)-based transformer schemes were used [36]. Diffusion models, on the other hand, learn the actual data distribution through likelihood-based training, making them more interpretable as compared to adversarial networks. Therefore, researchers comprehensively evaluated four GANs (progressive GAN, StyleGAN 1–3) and a diffusion model using two segmentation networks, U-Net and a Swin transformer, for the task of brain tumor segmentation [37]. To address the issue of inter-class and intra-class problems, a gated global–local attention (GGLA) mechanism was developed. The gating mechanism within the GGLA dynamically balances the contributions of global and local information, enabling the model to adaptively focus on the most relevant features for accurate classification. Additionally, an enhanced super-resolution generative adversarial network (ESRGAN) was coupled to generate images that balanced the MRI image data [38]. The cutting-edge techniques in multiple stages within a deep image recognition generative adversarial network (DIR-GAN) reported the robustness of brain tumor detection and classification in MRI scans [39]. Similar work was published [40] where HARA-GAN was proposed by integrating residual U-Net with hybrid attention and a relative average discriminator to mitigate noise caused by low undersampling rates. The results indicated that HARA-GAN outperforms DAGAN, RefineGAN, and RSCA-GAN methods based on error maps and quantitative evaluation metrics in terms of both image quality and consistencies on an MRI brain dataset. To improve on capturing long-range dependencies and spatial variations, Lyu et al. [41] reported a residual attention U-shaped network (RAUNet) for brain tumor segmentation which leverages the robust feature extraction capabilities of U-Net and the global context awareness provided by transformers to improve segmentation accuracy, and Ahmed et al. [42] introduced hybrid federated adversarial MRI enhancement (FAME) by integrating advanced GAN architectures such as multi-scale convolutions, attention mechanisms, and GNNs. Furthermore, self-generating few-shot brain tumor segmentation models like CDSG-SAM are also part of the literature [43] where a dynamic fuzzy support mask decoder module (DFSMD) was used within them to enhance the classification accuracy of BTs.

Despite the cited advancements in deep learning for brain tumor classification, a critical research gap persists in the generation and diversity of synthetic MRI images. Existing studies largely focus on the aspect of classification using CNNs, transformers, and hybrid architectures leading to sub-optimal generalization in terms of high-quality and diverse tumor representations. The aim of this study is to propose a dual-stream generator architecture in GANs which incorporates contrastive learning and multi-stream feature fusion for enhancing the diversity and realism of synthetic MRI images. The present research is focused on leveraging dual-stream generator frameworks to ensure a balanced image synthesis.

The dual-stream generator and three discriminators were designed within an adversarial framework to enhance competitive learning. Traditional GAN-based augmentation suffers from mode collapse due to separate generators, a limitation addressed by our dual-stream architecture. The integration of a contrastive latent projection (CLP) module preserves semantic consistency in augmented images, while contrastive learning ensures diverse yet coherent feature representations. We propose a novel objective function for DSCLPGAN, optimized for discriminative feature learning. Performance evaluation using assessment metrics demonstrates superior diversity and generalization in the generated images.

The main contributions of this paper are summarized below:

- The proposed dual-stream augmentation framework utilizes a single generator with dual perturbations to enhance realism and diversity by effectively capturing both local and global variations in medical images.
- A rigorous mathematical formulation is developed, incorporating a CLP module to preserve semantic integrity and enhance model generalization in image augmentation tasks.
- A three-discriminator architecture is introduced, operating in parallel to assess image quality, diversity, and frequency consistency. Additionally, D1 performs classification, eliminating the need for a separate brain tumor (BT) classifier network.

## 2. Materials and Methods

A dual-stream single generator with CLP is an efficient, robust, and medically meaningful way to augment MRI images while ensuring high quality and diversity in synthetic images.

### 2.1. Dual-Stream Generator of Our Proposed Model (DSCLPGAN)

**Figure 1:**
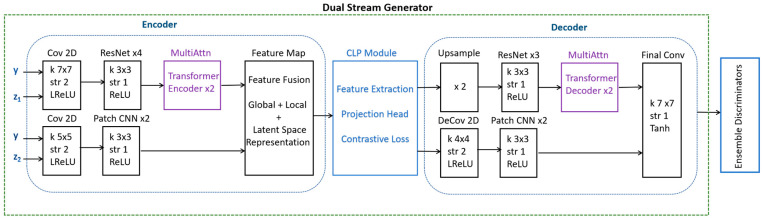
An illustration detailing the operations and blocks with an encoder, CLP module, and decoder of a dual-stream generator.

Instead of using two separate generators, a single generator handles both augmentation streams. A single generator with dual perturbations avoids image similarity issues generated by two generators by maintaining diversity within one network. In our model, one stream applies local variations and the other handles the global variations. An encoder extracts the latent representation from the input MRI scan. The latent space is split into two parallel streams, with one to emphasize localized variations and the second to emphasize global changes, as illustrated in Figure 1.

As illustrated in Figure 1, the dual-stream generator uses a CNN/transformer encoder for the extraction of global features and a patch-wise CNN for learning the local features within an image. This study uses DSCLPGAN, which is designed to generate high-quality synthetic MRI brain images. The method employs a dual-stream generator along with three discriminators, each serving a distinct role in improving the fidelity and clinical utility of generated images. The model was trained on a publicly available MRI dataset from Kaggle, which comprises 3064 T1-weighted contrast-enhanced images categorized into three tumor types: meningioma (708 slices), glioma (1426 slices), and pituitary tumor (930 slices). The dataset used in this study consists of T1-weighted contrast-enhanced MRI slices. The generator incorporates two parallel streams to synthesize high-quality MRI images. The content stream captures the structural and spatial information of brain tumors and the texture stream focuses on enhancing fine details and contrast, ensuring realistic appearance in synthetic images. The outputs of both streams are fused via CLP to enforce feature alignment between real and synthetic images. The final synthesis is performed using a shared decoder that reconstructs high-resolution MRI images by integrating the complementary information from both streams. Three discriminators are employed to enhance the robustness of the network.

The CLP module applies contrastive learning to project real and fake features into a contrastive latent space to ensure that the augmented images remain semantically close to the original. It applies contrastive learning using a similarity-based loss function as given in Equation (1).

(1) $$L_{CLP}=-log\frac{exp(sim(Z_{1},Z_{2})/\tau )}{\sum_{j}exp(sim(Z_{1},Z_{j})/\tau )}$$

where sim (.) is the cosine similarity between z_1_ and z_2_, and z_1_ and z_2_ are the embeddings of the anchor and the positive sample (similar samples), whereas z_j_ represents the negative samples. τ is the temperature parameter controlling how strongly differences are penalized. The value of cosine similarity ranges from −1 to 1. For contrastive learning, we tried to maximize the cosine similarity between positive pairs and minimize it for negative pairs.

Equation (1) aims to pull positive pairs closer in the embedding space and push negative pairs further apart. The denominator is the sum of the positive terms and the sum of the negative terms. For each sample, we compare it against every other sample in the batch. The negative samples contribute negatively to the probability of the anchor being close to the positive pair. This encourages the network to increase the distance between the anchor and negative samples. The logarithmic function in the loss is used to convert the ratio into a loss that can be minimized. By maximizing the numerator and minimizing the denominator (which includes the negatives) in Equation (1), the model learns to distinguish positive pairs from negative pairs.

**Figure 2:**
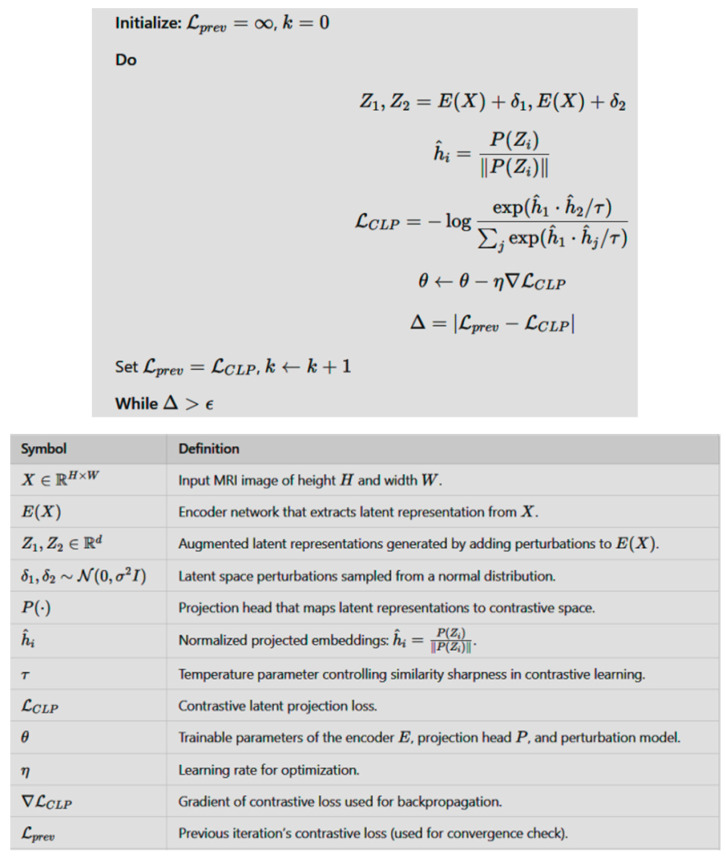
The do while code of CLP with the input MRI image (X), encoder, and projection head along with a description of the involved parameters.

The do while pseudocode representation of CLP with the input MRI image (X), encoder, and projection head with perturbations is given in Figure 2 along with a description of the involved parameters.

The dual-stream decoder reconstructed the image using the global Reconstruction Path, local refinement path, and skip connections as illustrated in Figure 1. The global path exploited the ResNet/transformer decoder and the local path involved the Deconv and CNN blocks. The final output included high-quality MRI images with structure and texture accuracy.

### 2.2. Complete Architecture of Our Proposed Model (DSCLPGAN)

**Figure 3:**
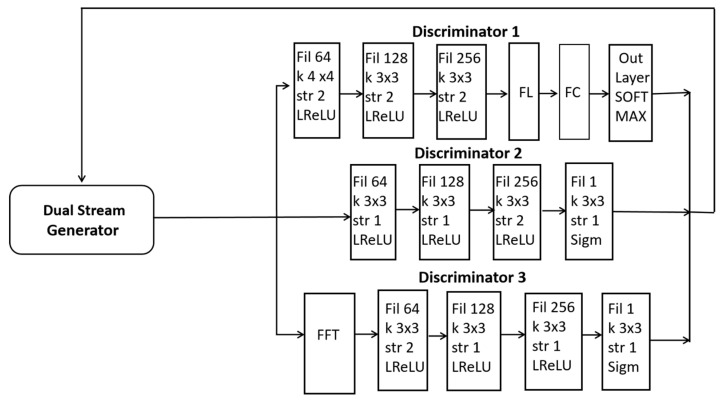
A schematic illustration of our proposed dual-stream generator along with three discriminators.

In this paper, a dual-stream generator and triple discriminators are used for the adversarial training of diverse synthetic images as presented in Figure 3. The first discriminator D_1_ (global discriminator) is for the classification of BTs. The second discriminator D_2_ (local discriminator) is designed to analyze small patches within an image including specific tumor regions. The third discriminator D_3_ converts images to frequency space for global consistency. A detailed schematic including the details of the CNN and layers is presented in Figure 3.

### 2.3. Mathematical Formulation

The dual-stream generator (G) consists of a spatial stream G_S_ (z) to generate spatial features. The latent contrastive stream G_L_ (z) enforces latent space similarity via CLP. The generator learns to map latent noise to a realistic MRI image as shown in Equation (2).

(2) $$x^{\prime }=G\left(z)=G_{s}(z)+G_{l}(z\right)$$

where $G_{s}(z)$ generates high-resolution spatial details and $G_{l}(z)$ enforces feature consistency.

D_1_ learns to classify both the real and generated MRI images into the medical conditions given in Equations (3) and (4).

*p* (y_r_∣x_r_) = D_1_(x_r_) (3)

*p* (y′∣x′) = D_1_(G(z)) (4)

where y_r_ is the ground truth class.

The classification loss is governed by Equation (5).

(5) $$L_{cls}=-E_{\sim P_{data\left(x\right)}}\sum_{i=1}^{c}{y^{i}}_{r}logD_{1}^{i}\left(x_{r}\right)$$

where c represents the number of classes and ${y^{i}}_{r}$ is the ground truth class indicator.

The local D_2_ discriminator evaluates patches from real and fake MRI images and the adversarial loss is given by Equation (6).

(6) $$L_{D2}={-E}_{x_{r}\sim P_{data\left(x\right)}}\left[\log D_{2}\left(x_{r}\right)]+E_{z\sim P_{z\left(z\right)}}[log({1-D}_{2}(G\left(z\right)))\right]$$

MRI scans are evaluated in the Fourier domain by D_3_ to ensure frequency consistency and the Fourier adversarial loss is computed using the expression in Equation (7).

(7) $$L_{D3}={-E}_{x_{r}\sim P_{data\left(x\right)}}\left[\log D_{3}\left(F(x_{r}\right))]-E_{z\sim P_{z}}[log({1-D}_{3}(F\left(G(z\right))))\right]$$

The loss function is the aggregate loss total of all three discriminators, CLP loss and the hyper-parameters balancing each loss term and is given by Equation (8).

(8) $$L_{D}=\lambda_{cls}L_{D1}+{\lambda_{adv}L}_{D2}+\lambda_{freq}L_{D3}+\lambda_{clp}L_{clp}$$

The objective of training is to minimize the generator loss and to maximize the discriminators loss as indicated in Equation (9).

(9) $${G^{*}=argmin}_{G}{{max}_{D}L}_{G}$$

where $L_{G}=\lambda_{cls}L_{D1}+{\lambda_{adv}L}_{D2}+\lambda_{freq}L_{D3}+\lambda_{clp}L_{clp}$.

The ultimate objective is to produce MRI images that appear realistic at the global level with accurate and realistic local details and further maintain semantic consistency with the real image.

## 3. Results and Analysis

The brain tumor dataset used (Figure Share 2024) contains 3064 T1-weighted contrast-enhanced images with three types of brain tumor: meningioma (708 slices), glioma (1426 slices), and pituitary tumor (930 slices). The 5-fold cross-validation indices are also provided. Prior to augmentation and the training phase, the MRI scans were subjected to a series of pre-processing stages for dataset standardization. Using our proposed DSCLPGAN architecture, the image data in each class were amplified by a factor of 75 to increase the diversity and to validate the generalization ability of our proposed network. We tested our GAN-generated image data underlying objective to underscore the impact and extent of image augmentation in improving diversity and generalization. All the models were implemented using Pytorch™. The four possible output labels are 0—no tumor, 1—meningioma tumor, 2—glioma tumor, and 3—pituitary tumor. The connection processing of research results in our dual-stream contrastive latent learning GAN for brain image synthesis was established through multiple quantitative and qualitative evaluations. For this purpose, SSIM loss in the revised version quantitatively verifies structural integrity by measuring perceptual similarity between generated and ground truth images. Our results indicate lower FID values compared to traditional augmentation methods, confirming that our mathematical framework enhances the realism and diversity of generated images. The contrastive latent learning component in the revised version enforces separation between meaningful variations while preserving critical pathological features and this is reflected in our latent space distance plots against training epochs, which show progressive clustering of similar image representations while maintaining distinct variations. Further, by varying augmentation diversity levels, we observed a direct impact on classification performance, indicating that our generated samples effectively contribute to model generalization in the revised version. For instance, higher augmentation diversity leads to improved classification accuracy and contrastive learning improves task-specific learning. PSNR trends show consistent improvement across epochs, suggesting that the network progressively enhances image fidelity.

**Figure 4:**
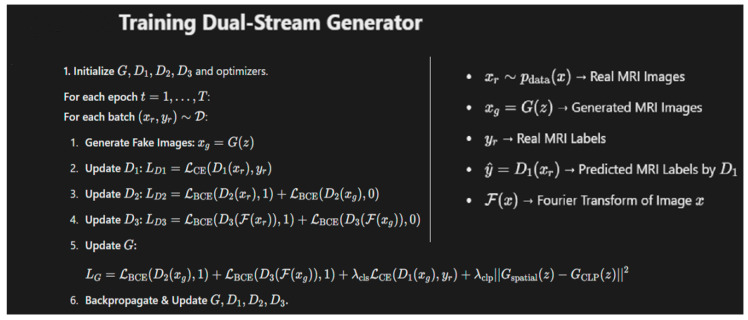
The algorithm used for training the dual-stream generator in our proposed model along with details of the key symbols used.

A latent dimension of 100 and batch size of 64 was selected for 50 epochs with a learning rate of 0.0001. The Adam optimizer was used for optimization with a β_1_ value of 0.5 and β_2_ of 0.999. L_3_ Regularization was applied to the weights of the network to penalize large weights. The training process is shown in Figure 4 in which the dual-stream generator is trained by simultaneously optimizing two streams: one generating realistic augmented images and the other enforcing latent space consistency. An adversarial loss helps the generator produce high-quality outputs, while a reconstruction loss maintains fidelity to the original data.

**Figure 5:**
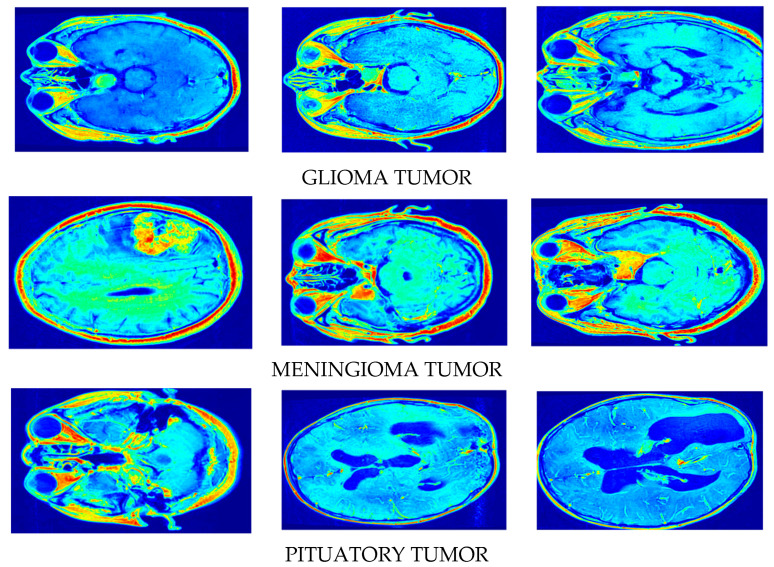
Synthetic image creation via our proposed DSCLPGAN for different brain tumors.

A few sample images generated by our model using the original MRI images are presented in Figure 5. Our algorithm architecture involves two networks/streams that process the input data in parallel: the input stream and the latent space stream. The input stream takes in real brain images for synthesis and the latent space stream generates informative features that guide image synthesis. The goal of contrastive learning is to ensure that similar brain images remain close in the latent space, while dissimilar images at distance. The process of how informative features enter the processing of synthetic patterns is presented below.

The initial data input of MRI images is transformed by our algorithm into a latent representation. The encoder captures key features like shapes, textures, and other discriminative patterns from the MRI image and generates a latent representation of a synthetic image. After obtaining the latent representations of both the real and synthetic images, a contrastive loss is applied. The objective is to pull similar latent representations (e.g., tumor types or healthy brain regions) closer together and push dissimilar ones further apart. The latent features in this step are informative features that help in distinguishing between different tumor types or between healthy (positive pair) and affected brain tissue. The network uses these latent features to guide the learning of both the synthesis of realistic images and classification tasks. The latent representations generated above are fed into the generator network to synthesize realistic brain images that match the informative features of the input images. The latent vectors produced in the contrastive learning process serve as a conditioning mechanism, ensuring that the generator produces images with accurate anatomical details and tumor patterns based on the learned representations. The image discriminator evaluates whether the generated synthetic image is realistic (based on the real data distribution) or fake. During training, the generator improves its ability to synthesize images that look realistic, while the discriminator becomes better at distinguishing real from fake images. This process helps guide the generator to produce realistic brain images with tumors that match the real distribution of brain image data. Discriminator 1, which is specifically designed for tumor classification, uses the generated synthetic brain images or the latent representations to classify whether the image contains a tumor or not. The latent representations (or the synthetic images themselves) are used as input to this discriminator. Since the generator has been trained to capture key features related to the tumor’s structure in the latent space, the tumor classification discriminator can more effectively identify the presence of a tumor. Discriminator 2 (image discriminator) guides the generation of realistic brain images and Discriminator 3 (latent discriminator) ensures that the latent space maintains the necessary structure for distinguishing between different brain regions and tumor types.

**Figure 6:**
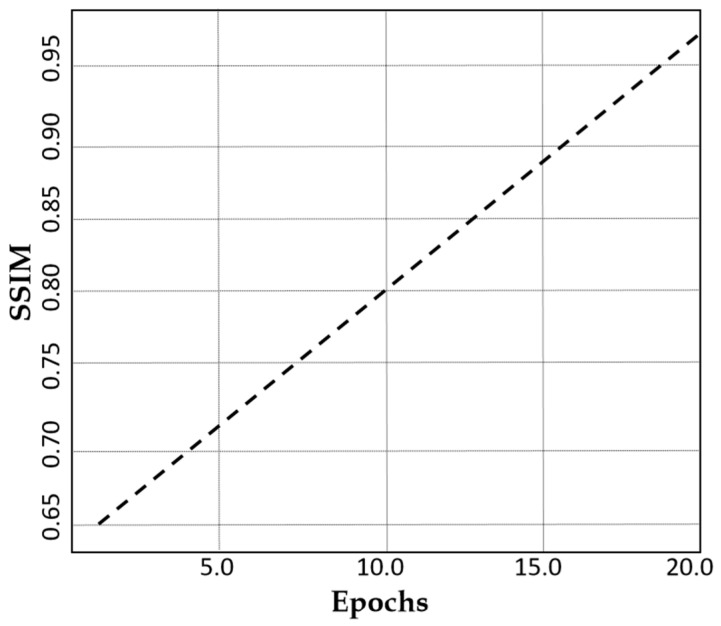
A plot of SSIM against the number of epochs.

The Structural Similarity Index Measure (SSIM) loss shows a remarkable improvement from 0.65 to 0.99 over 20 epochs as indicated in Figure 6. This demonstrates the superior structural quality of our dual-stream generator with CLP, hence ensuring that augmented MRI images remain highly realistic and clinically valuable. Such a high SSIM score reinforces the robustness of our method in generating high-quality medical images.

**Figure 7:**
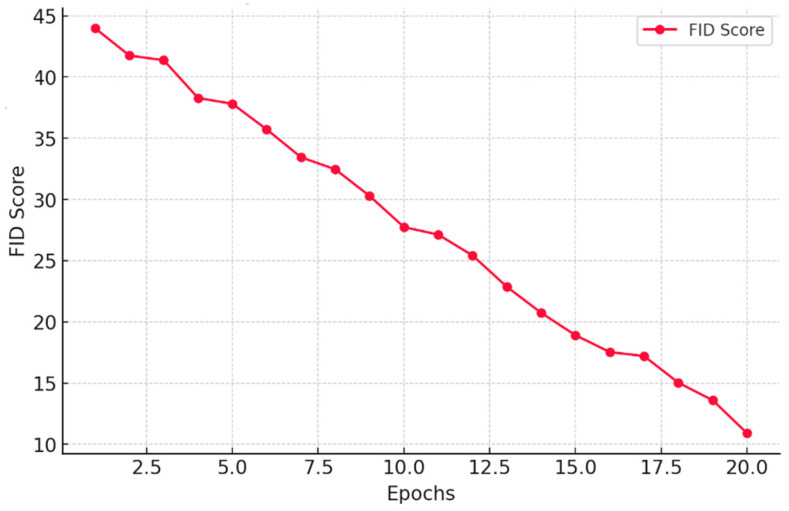
A plot of FID score against the number of Epochs.

Similarly, the Fréchet Inception Distance (FID) in Figure 7 demonstrates a remarkable decline from 45 to 12 over 20 epochs. This signifies that our model progressively refines image quality, making synthetic data perceptually closer to real MRI scans. Such a low FID score highlights the effectiveness of our method in preserving critical medical features while ensuring diverse and realistic augmentation.

**Figure 8:**
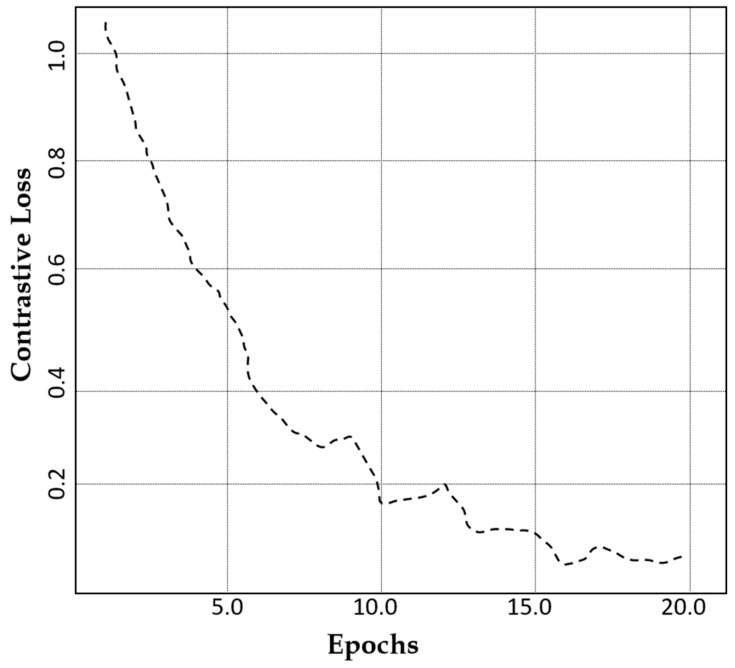
A comparison of the contrastive loss of our model against the number of epochs.

The contrastive loss exhibits a value of 0.97 after 20 epochs as indicated in Figure 8. This reduction signifies improved feature consistency, ensuring that the generated images retain essential medical characteristics without losing diversity.

**Figure 9:**
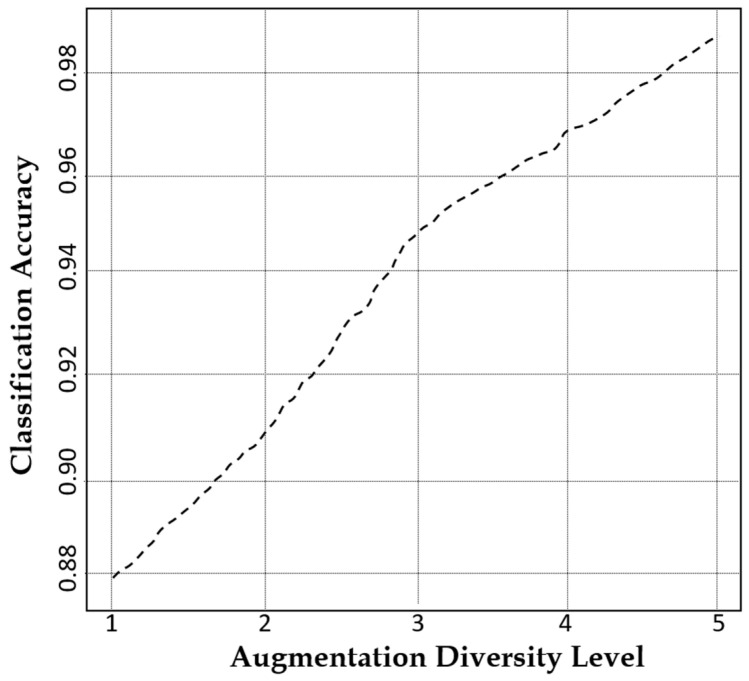
A plot of the classification accuracy of our model against augmentation diversity level.

The augmentation diversity level refers to the degree of variation introduced in synthetic data to enhance model generalization while preserving essential structural features. At an augmentation diversity level of 5, our model achieves an outstanding classification accuracy of 99.76% as illustrated in Figure 9, proving its unparalleled ability to generate diverse yet highly realistic MRI images. This exceptional performance is attributed to the CLP module in our model for ensuring meaningful variations.

**Figure 10:**
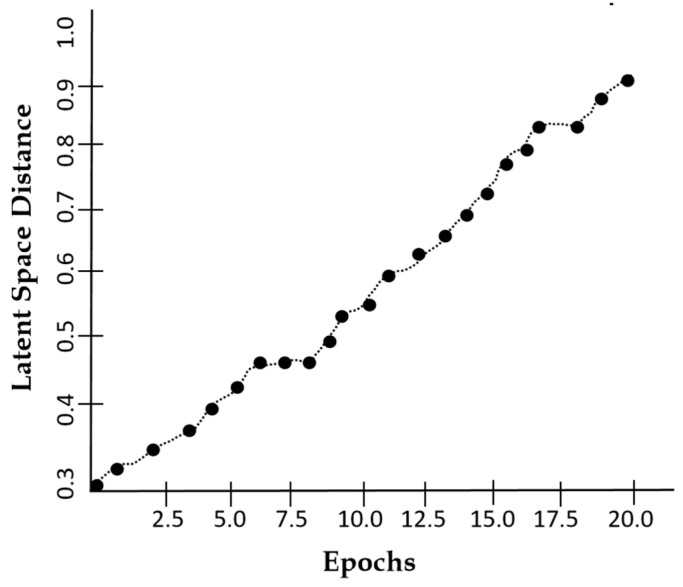
A plot of latent space distance against epochs using DSCLPGAN for feature distinction.

**Figure 11:**
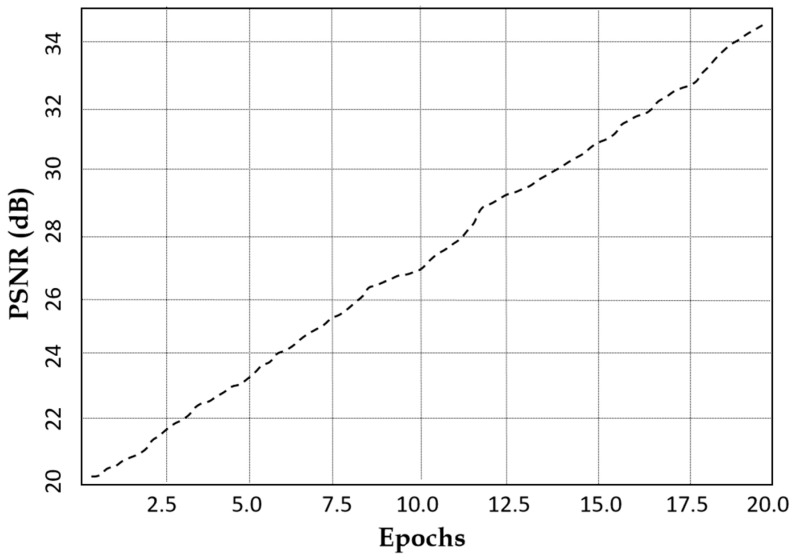
Peak signal-to-noise ratio characteristics of our model against the number of epochs.

Figure 10 suggests that the latent space distance shows an upward trend with the number of epochs which reflects the model’s ability to enhance feature distinction. The latent space distance ensures that the generated MRI images are not only diverse but also structurally coherent, preventing redundancy and mode collapse. Our model achieves a PSNR of 34.6 dB within 20 epochs as indicated in Figure 11, suggesting that our augmented images closely resemble the real MRI scans.

To highlight the effectiveness of the proposed DSCLPGAN, a comparative analysis was conducted against state-of-the-art GAN-based and transformer-based augmentation models and is illustrated in Table 1. The evaluation considers key performance metrics such as SSIM, PSNR, and FID score. The models selected for comparison included BIGGAN [44], MAGE [45], TransGAN [46], SR TransGAN [47], CTGAN [48], StyleGANv2 [49], SFCGAN [50], VQ-GAN [51], and 3D Pix2Pix GAN [52]. An inter-comparison with such models with benchmark performance characteristics underscores the efficiency of DSCLPGAN in generating high-quality and diverse medical images.

## 4. Conclusions

In this study, we introduce a novel approach for generating synthetic MRI brain tumor images using a dual-stream generator fused GAN via CLP. This technique helps maintain the structural accuracy of the images while also boosting performance for tasks like classification. Our experiments show that the model works well across various evaluation measures, such as SSIM, FID, contrastive loss, and latent space consistency, demonstrating its ability to generate high-quality and realistic images that closely match the original ones. A key feature of our model is the use of triple discriminators, which assess the images from different angles—evaluating both their realism and their consistency in latent space. This multiple-level scrutiny ensures that the images not only look real but also make sense in the context of the underlying data, which is particularly important for medical imaging applications. The results are indicative of the fact that our model is better at generalizing. This ability to generalize is imperative in real-world medical settings where data can vary significantly. In addition to its success in MRI-based applications, the model carries great potential for broader use in other clinical imaging fields for informed decision-making.

## Figures and Tables

**Table 1:** A comparison of our proposed model (DSCLPGAN) with state-of-the-art methods for image synthesis.

Method	SSIM	FID	PSNR
BIGGAN [44]	0.7314	47.63	25.89
MAGE [45]	0.8220	45.62	27.28
TransGAN [46]	0.8376	35.45	27.66
SR TransGAN [47]	0.8504	31.29	30.28
CTGAN [48]	0.8755	29.10	26.47
StyleGANv2 [49]	0.8841	32.56	29.31
SFCGAN [50]	0.9077	28.04	29.14
VQ-GAN [51]	0.9166	26.55	31.04
3D Pix2Pix GAN [52]	0.9210	27.87	30.19
Proposed DSCLPGAN	0.9861	12	34.6

## Data Availability

Data supporting reported results is available on request.
